# Long-term follow-up of bone remodelling after cementless hip arthroplasty using different stems

**DOI:** 10.1038/s41598-020-67189-x

**Published:** 2020-06-23

**Authors:** Steffen Brodt, Georg Matziolis, Bettina Buckwitz, Timo Zippelius, Patrick Strube, Andreas Roth

**Affiliations:** 1 Orthopaedic Professorship of the University Hospital Jena, Orthopaedic Department Waldkliniken Eisenberg, Klosterlausnitzer Str. 81, 07607 Eisenberg, Germany; 2 University Hospital Jena, Department of Trauma-, Hand- and Reconstructive Surgery, Am Klinikum 1, 07747 Jena, Germany; 3 University Hospital Leipzig, Clinic of Orthopedics, Traumatology and Plastic Surgery, Department Arthroplasty/Orthopedics, Liebigstr. 20, 04103 Leipzig, Germany

**Keywords:** Musculoskeletal system, Bone, Outcomes research

## Abstract

The present paper is concerned with the investigation of the phenomenon of long-term bone remodelling on cementless hip replacements. Changes in bone density in the periprosthetic region around the stem, measured by dual X–ray absorptiometry (DXA), were used as a measure of the osseous adaptation reaction. A postoperative follow-up of the use of four different types of prostheses of varying design after on average 13.3 (11.4–14.5) years. Specifically, the prostheses assessed in this study were the CLS/Spotorno stem with the Allofit cup by Zimmer, the Vision 2000 stem with the Duraloc cup by DePuy Synthes, the AlphaFit stem with the AlphaLock cup by Corin and the Mayo stem with the Trilogy cup by Zimmer. For the DXA measurement, the femur was divided into the zones suggested by Gruen *et al*. On the femur, there was a significant reduction in bone mineral density (BMD) in the proximal Region Of Interest (ROI) 1 (p = 0.003) and 7 (p < 0.001), whilst there was a significant increase in ROI 4 (p = 0.03). A greater degree of bone atrophy was seen in patients aged 60 years and older and in female patients. A remarkable finding when comparing the stems was a significantly greater reduction in BMD in ROI 6 (p = 0.003) in the case of the Vision 2000 stem and a markedly, but not statistically significantly smaller reduction in BMD in ROI 7 (p = 0.18) in the case of the short-stem Mayo-type prosthesis. The best clinical results were found with the use of the latter. The investigations provide a starting point for establishing a differential indication in the choice of prosthesis types, depending on age and sex, the use of short-stem prostheses, as well as the administration of bone-effective drugs for the prevention of stress shielding.

## Introduction

After implantation of a cementless total hip replacement, the surrounding bone stock reacts to the implant and the changed biomechanics in a characteristic way. This change can be quantified by using DXA to measure bone density and has already been well documented over the short and medium-term postoperative course. The greatest loss in measured bone mineralisation relative to the baseline density, in the sense of stress shielding, takes place especially in the first 6–12 months, mainly in the Gruen zones 1 and 7^1–5^. At the same time, there are marked differences in bone demineralization between different prosthesis types. Stems with proximal load transmission appear to cause less stress shielding in the short to medium term^3,6,7^. Female sex and a low systemic bone density also appear to promote calcar atrophy^8,9^.

In contrast, the study results concerning bone remodelling of the femur over the long-term course are inconsistent. Some authors report a demineralisation of the periprosthetic bone stock, also occurring over the long term^4,8,10,11^.

For long-term reliable osseous integration of the prosthesis and for the prevention of periprosthetic fractures after bagatelle trauma, sufficient sustainable periprosthetic bone quality is needed. Therefore, it is important to document long-term osseous remodelling process around different prosthesis types, to allow for a better assessment of potentially differential indications and future prosthesis designs.

The objective of the present study was to demonstrate the reaction of the femoral bone to different stems over a follow-up period of at least 11 years using DXA. Both long-term changes in bone density of the individual implants and corresponding differences across prosthesis types were to be analysed. A standardised patient interview was conducted to assess functionality and patient satisfaction.

## Material and methods

**Figure 1:**
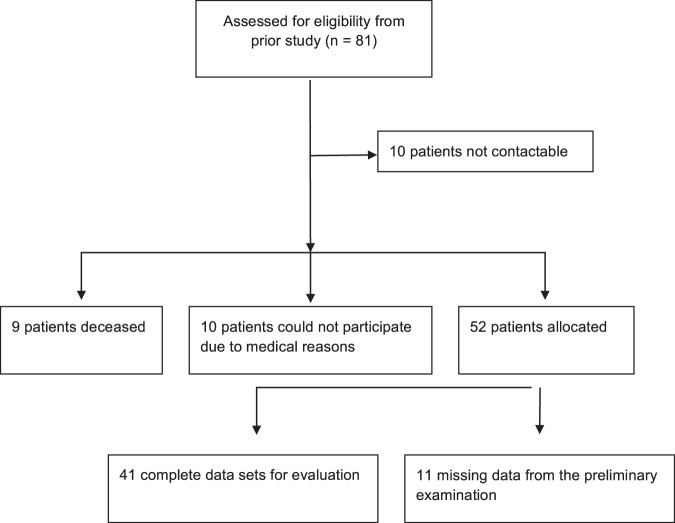
Flow Diagram.

81 patients with cementless hip replacements were invited by mail and by telephone to undergo an examination (Fig. 1). This patient population had undergone arthroplasty by the same surgeon within the context of an earlier study between 1999 and 2002 and had already been subject to a follow-up study after one year by Roth *et al*. (2005)^3^. Study approval was obtained from the ethics committee of the University Hospital Jena, Germany (No. 3817-07/13) and all methods were performed in accordance with the relevant guidelines and regulations. All patients were informed about the study preoperatively and gave their written informed consent to participate in the study.

The indication for implantation of a hip replacement was primary osteoarthritis of the hip in 54.3% of the cases and secondary osteoarthritis of the hip in 45.7% of the cases.

A total of 52 patients attended the follow-up examination. For 11 of those patients, no earlier data on pre- and early postoperative bone density measurements were available in our databases. The remaining 41 patients (50.6%) were included in our analysis. Of the 29 invited persons who did not participate in the follow-up examination, nine were deceased, 10 patients were unable to attend the examination due to non-hip-related health problems, and 10 patients had moved to a new address or did not react to the invitation.

The 41 patients, who attended our longer-term follow-up examination, were on average 56.9 (±9.0) years old at the time of surgery. Our examination took place on average 13.3 (±0.7) years after the initial surgery. The ratio of men to women among our study subjects was 1:3 (Table 1).

**Table 1:** Number of prostheses followed up according to type.

Prosthesis type	Stem	CLS	Vision 2000	AlphaFit	Mayo	Total(median)
	Cup	Allofit	Duraloc	AlphaLock	Trilogy	
Number of patients, initial population		26	29	18	8	81
Number of patients, current		15	11	8	7	41
male		2	1	5	3	11
female		13	10	3	4	30
Proportion of retraced patients in %		57.7%	37.9%	44.4%	87.5%	50.6%

**Figure 2:**
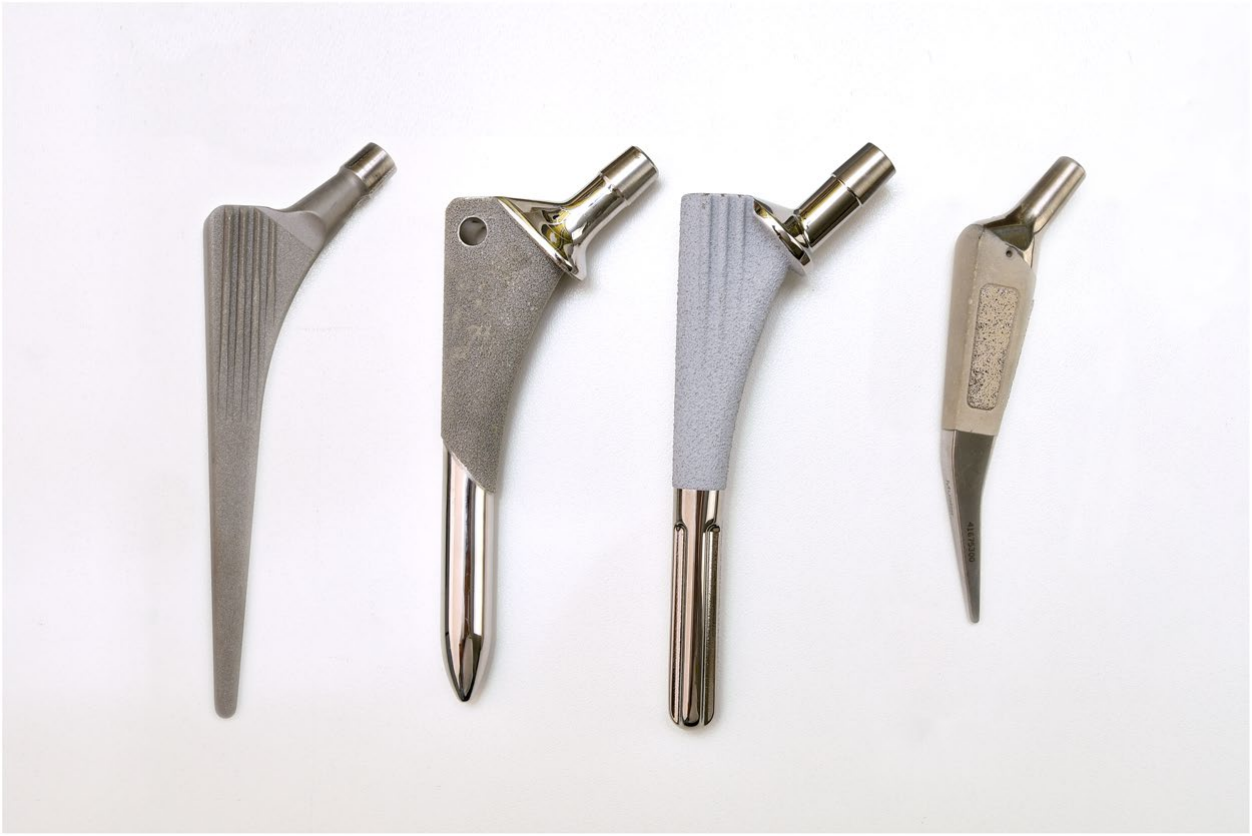
CLS Spotorno stem, Vision 2000 stem, AlphaFit stem and Mayo stem (from left to right).

Four types of cementless hip replacement commonly implanted at the time of the operation were used and compared with each other. These implants were the CLS/Spotorno stem with the Allofit cup (Zimmer Biomet Inc, Warsaw, IN, USA), the Vision 2000 stem with the Duraloc cup (DePuy Synthes, Warsaw, IN, USA), the AlphaFit stem with the AlphaLock cup (Corin PLC, Cirencester, England) and the Mayo stem with the Trilogy cup (Zimmer Biomet Inc, Warsaw, IN, USA) (Fig. 2).

### Stems

The CLS stem has been routinely used for 25 years and is still used regularly today. It is made of a titanium alloy. It has a conical shape both in the sagittal and in the frontal plane. The surface is corundum blasted. It has ribs running longitudinally along its proximal section^12,13^. Long-term stability is achieved by bony ongrowth to the implant surface. In this type of stem, we find a metaphyseal load-transmission.

The Vision 2000 stem consists of a cobalt-chrome alloy. It has a cone-like shape distally and is coated osteoinductively only in the proximal half^14^. The load-transmission in this type of stem is meta-diaphyseal, and therefore a bit more distal than we find it in the CLS stem.

The AlphaFit stem is a titanium stem coated porously in the proximal half, with longitudinal macrostructuring. The stem tip is uncoated and polished. The load-transmission in this stem is also meta-diaphyseal and similar to that in the Vision 2000 stem.

The Mayo stem is an anatomical short-stem prosthesis with a metaphyseal load-transmission. It is made of titanium and is double-wedge shaped proximally. The distal end of the prosthesis is angled and is not designed for prosthesis fixation, but for correct positioning^15^. The characteristics of the different stem types are described in Table 2.

**Table 2:** Characteristics of the different stem types.

Stem	Alloy	Anchoring	shape	surface design
CLS Spotorno	titanium	metaphyseal	conical in frontal and lateral plane	corundum blasted
Vision 2000	cobalt-chrome	meta-/diaphyseal	straight, macro-structure	proximal Porocoat®, distal polished
AlphaFit	titanium	meta-/diaphyseal	straight, macro-structure	proximal calcium phosphate coated, distal polished
Mayo	titanium	metaphyseal	short-stem, double wedge-shaped	proximal macro-structure with fibre-mesh

### Bone density measurement

DXA scans were performed on the acetabulum and the proximal femur of the side treated with the hip replacement. The device used was the QDR 45000 W Hologic (Waltham, MA, USA), which was also used for the measurements one year after the surgery. The device’s metal-removal software was used in all postoperative measurements. The bone density values obtained were stated in g/cm^2^.

**Figure 3:**
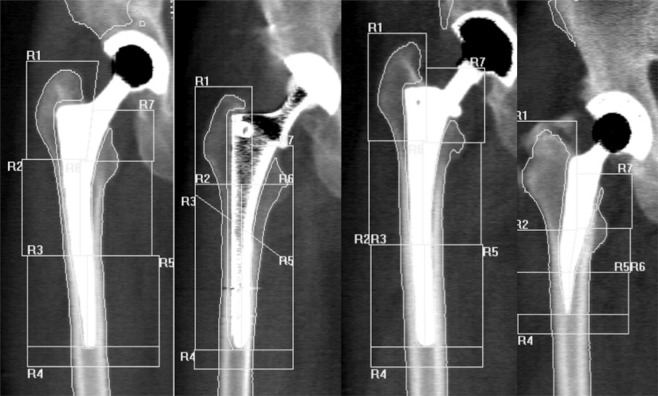
Position of the ROIs on the CLS Spotorno, Vision2000, AlphaFit and Mayo stems (from left to right).

The proximal femur was divided into 7 regions of interest (ROIs) based on the classification according to Gruen^16^ (Fig. 3).

The average follow-up period was 13.3 years (median 13.5 years; 11.4–14.5 years) after surgery. In addition, for comparison, the raw data of the preoperative and immediately postoperative measurements on the above-mentioned regions of the respective patients were recovered from the archive and, to prevent a systematic error, re-evaluated along with the current measurements.

### Clinical evaluation

All of the patients who appeared for the follow-up examination were examined clinically and asked to state their level of satisfaction with their clinical outcome. Beside the medical history and examination, the clinical evaluation also included the Harris Hip Score and WOMAC for better comparability.

### Statistical analysis

In the statistical analysis, both the arithmetic mean and the median were calculated and the respective standard deviation was stated for all variables. To test for group differences, the Mann-Whitney U test for nonparametric and independent samples was used for inter-individual comparisons of the implant types with each other. The Wilcoxon test for nonparametric and dependent samples was used to test for intra-individual differences, i.e., differences over time in the same individual. The level of statistical significance was set at 0.05 for all tests.

## Results

### Bone density measurements on the femur

Considering all implant types together, comparison of the early postoperative DXA scans with those after a longer-run follow-up after at least 11 years showed a statistically significant reduction in bone density in the two proximal ROIs 1 and 7 as well as 5 and 6 (p < 0.05) and a significant increase in bone mineral density (BMD) in ROI 4 (p < 0.05). A reduction of −0.070 g/cm^2^ (−10.11%, p = 0.003) was demonstrated in ROI 1, and of −0.254 g/cm^2^ (−25.33%, p < 0.001) in ROI 7. A reduction of −0.155 g/cm^2^ (−12.30%, p < 0.001) was seen in ROI 6. Very small but statistically significant changes were found in ROIs 4 and 5 [ROI 4: +0.025 g/cm^2^ (1.58%, p = 0.03), ROI 5–0.006 g/cm^2^ (−0.35%, p = 0.048)]. There was a nonsignificant reduction in bone density in ROI 2, at −0.063 g/cm^2^ (−5.03%), and no major change in ROI 3, at +0.005 g/cm^2^ (0.34%).

### Differences between the stem types

**Figure 4:**
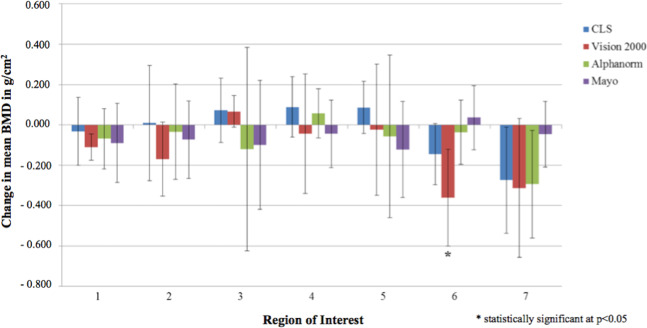
Change in the mean BMD on the femur at follow-up compared with the postoperative baseline value according to ROI and prosthetic stem (*denotes significant changes).

The inter-individual comparisons of the stem types among each other revealed a statistically significantly stronger reduction in bone density for ROI 6 for the Vision 2000 stem, at −0.360 g/cm^2^ (−29.92%, p = 0.003) (Fig. 4).

Overall, the Vision 2000 stem showed the strongest reduction of BMD in ROIs 1 and 7. In ROI 1 the reduction was −0.110 g/cm^2^ (−17.12%) and −0.313 g/cm^2^ (−33.04%) in ROI 7. There was no significant difference compared with the other stems at ROI 1 (p = 0.62) and ROI 7 (p = 0.18).

The AlphaFit stem showed a nonsignificant reduction of BMD. With a reduction in bone density of −0.293 g/cm^2^ (−29.76%) in ROI 7 and of −0.069 g/cm^2^ (−10.19%) in ROI 1, these differences tended to be slightly lower than in the Vision 2000 stem. In ROI 4, the bone density increased by 0.058 g/cm^2^ (+3.60%) without showing any significance.

A slight increase in bone density in ROI 4 was also found in the CLS stem [0.089 g/cm^2^ (+5.48%)]. In contrast to the first two stems above, an increase, which was not statistically significant, also occurred in regions 3 and 5 [0.073 g/cm^2^ (+4.74%) and 0.087 g/cm^2^ (+5.48%)].

The Mayo stem showed the lowest reduction in bone density in region 7 and thus the greatest deviation from the results of the other stems. With an average reduction in bone density in this region of −0.046 g/cm^2^ (−5.16%), compared with −0.313 g/cm^2^ (−33.04%) in the Vision2000, −0.293 g/cm^2^ (−29.76%) in the AlphaFit and −0.274 g/cm^2^ (−24.85%) in the CLS stem, a strong tendency was seen here, but this was not statistically significant. In the likewise proximally located ROI 1, values of −0.089 g/cm^2^ (−11.62%) were found, which were comparable to those in the stems already described above. Moderate reductions were again found in ROIs 2, 3 and 5 [−0.073 g/cm^2^ (−5.12%), −0.099 g/cm^2^ (−5.72%) and −0.121 g/cm^2^ (7.19%), respectively]. Deviating from all other stems, a slight increase in bone density, of 0.036 g/cm^2^ (2.65%) was found in ROI 6. On the prosthesis tip (ROI 4), a trend towards a decrease in BMD, of −0.044 g/cm^2^ (−2.68%), was observed (Table 3).

**Table 3:** Change in BMD (±SD) on the femur in g/cm^2^ and in % of the baseline value (*statistically significant at p < 0.05).

Model	ROI 1	ROI 2	ROI 3	ROI 4	ROI 5	ROI 6	ROI 7
CLS	−0.032 (±0.169)−4.41%	0.009 (±0.287) 0.76%	0.073 (±0.161) 4.74%	0.089 (±0.150) 5.75%	0.087 (±0.129) 5.48%	−0.145 (±0.152)−10.97%	−0.274 (±0.264)−24.85%
Vision 2000	0.110 (±0.066)−17.12%	−0.171 (±0.183)−14.05%	0.067 (±0.079) 5.01%	−0.044 (±0.297%)−2.91%	−0.023 (±0.325)−1.62%	−0.360* (±0.240)−**29.92%**	−0.313 (±0.344)−33.04%
AlphaFit	−0.069 (±0.151)−10.19%	−0.034 (±0.237)−2.75%	−0.120 (±0.505)−7.47%	0.058 (±0.121) 3.60%	−0.057 (±0.404)−3.35%	−0.037 (±0.160)−3.13%	−0.293 (±0.268)−29.76%
Mayo	−0.089 (±0.197) 11.62%	−0.073 (±0.192)−5.12%	−0.099 (±0.320)−5.72%	−0.044 (±0.168)−2.68%	−0.121 (±0.238)−7.19%	0.036 (±0.159) 2.65%	−0.046 (±0.163)−5.16%
all groups combined	−0.070* (±0.144)−**10.11%**	−0.063 (±0.237)−5.03%	0.005 (±0.277) 0.34%	0.025 (±0.255) 1.58%	−0.006 (±0.274)−0.35%	−0.155 (±0.225)−12.30%	−0.254* (±0.279)−**25.33%**

### Results of the WOMAC and harris hip score

In our longer-term follow-up examination, patients had the average of the global index of the WOMAC was 1.44 points, while the average of the Harris Hip Score was 85 points. In the WOMAC, the best average values were achieved by the Mayo/Trilogy prosthesis, with 0.69 points. At p < 0.014, these differed statistically significantly from those of the Vision 2000/Duraloc prosthesis, with 1.98 points. All of the other prosthesis pairings did not differ statistically significantly from each other (p > 0.05) (Table 4).

**Table 4:** Global index of the WOMAC and sum of the Harris Hip Score (*statistically significant at p < 0.05).

Score	CLS/Allofit	Vision2000/Duraloc	AlphaFit/AlphaLock	Mayo/Trilogy	total
WOMAC	1.29 (0.66)	1.98 (1.58)	1.69 (0.88)	0.69 (0.77)*	1.44 (0.94)
Mean ± SD	±1.29	±1.78	±2.14	**±0.50**	±1.54
Harris Hip Score Mittelwert (SD) Duraloc	85 (95)	88 (95)	85 (86)	91 (95)	85,00 (91)
Mean ± SD	±18.28	±18.63	±13.61	±8.23	±17.94

### Changes in BMD depending on sex and age

Considering all of the above prostheses, we established that women already showed lower bone density values in the postoperative measurement. However, this sex differences was only statistically significant in ROI 5 at our longer-term follow-up. There was virtually no sex difference in ROI 7 at longer-term follow-up (Table 5).

**Table 5:** Means of the postoperative BMD in g/cm^2^ on the stem according to ROI and sex (*statistically significant at p < 0.05).

Patient group	ROI 1	ROI 2	ROI 3	ROI 4	ROI 5	ROI 6	ROI 7
women	0.683	1.245	1.509	1.515	1.545^*^	1.258	1.003
men	0.728	1.264	1.573	1.682	1.691^*^	1.280	1.000

In contrast, longer-term bone density loss appeared to be more pronounced in women, since the bone density in ROI 1 as well as in ROIs 4–6 was significantly higher in the men in the follow-up measurement. The bone density in ROIs 2 and 7 also tended to be lower in female patients.

In addition, bone density development also varied by age of the patient at first implantation of the prosthesis. Here, too, only small differences between the age groups were found immediately postoperatively. Hence, stronger demineralisation at implantation in patients aged 60 years and older was only found over the long-term follow-up. Ten years postoperatively, a significantly lower bone density in ROI 1 was found in patients aged 60 years and older compared to patients below age 60 (p = 0.031). In ROIs 2–6, tendency towards stronger longer-run bone demineralisation with increasing age was also found, but without being statistically significant.

We also tested the correlation of Body-Mass-Index (BMI) and BMD. No statistically significant correlation could be determined here.

## Discussion

As the main result of the present study, a reduction in peri-implant bone density was seen in all of the cup and stem types investigated. The reduction in femoral bone density is influenced by various factors. Independently of the operation performed, a decrease in endosteal perfusion occurs as patients get older, leading to an age-related physiological bone atrophy with widening of the medullary space^17^. In a previous investigation, polyethylene wear from the inserts used and stress shielding have been identified the most important influencing factors for postoperative bone resorption after cementless hip arthroplasty^11^.

Stress shielding as a cause of the disproportionately large decrease in bone density around endoprosthetic implants, in contrast, has been the subject of many short- and medium-term studies. In these studies, a marked bone atrophy of between 10 and 30% was found, particularly in the proximal region of the prosthesis, which appeared to progress in most cases in the first and in part also in the second postoperative year^3,18^.

In line with results from other working groups, our investigations reveal further longer-term bone decalcification several years after surgery, especially in the calcar region of the femur around conventional cementless total hip replacements^4,19,20^. Reductions in bone density continue to be measurable beyond the first postoperative years. However, longer-term demineralisation varied markedly across individual regions, with no further demineralisation in some regions. This finding may be explained by the fact that, given stable osseointegration, sufficient load transmission takes place via the calcar and the greater trochanter over the longer term, including in the metaphyseal region. In the case of the short-stem prosthesis investigated, a remineralisation of the bone even occurred in the calcar region. Here, no pathological hypertrophy was detected in the distal ROI 4. For this reason, all of the prostheses examined must assume a more proximal load transmission, however in different degrees. These effects were most pronounced with the short stem and the CLS prostheses. The only cobalt-chrome stem in this investigation showed a trend towards the strongest bone atrophy after 13 years. This may have been partially caused by the greater stiffness compared with the titanium stems. Demographic factors associated with stronger bone atrophy on the femur are the female sex and an advanced age of the patient at the time of implantation of the prosthesis^21^.

In clinical practise, a tendency towards the short-stem prosthesis was seen. Whether this advantage was influenced by the lower degree of stress shielding or by other factors cannot be clarified by the present study.

A lesser degree of bone atrophy may prove to be advantageous in later revision operations and lower the risk of periprosthetic fractures.

Overall, the phenomenon of stress shielding can still be seen more than 10 years after cementless hip arthroplasty. Its extent depends on both the type of implant used and patient-related influencing factors such as age and sex.

## Data Availability

Data available from the corresponding author.

## References

[1] Spittlehouse AJ, Smith TW, Eastell R (1998). Bone loss around 2 different types of hip prostheses. J Arthroplasty.

[2] Aldinger PR (2003). Pattern of periprosthetic bone remodeling around stable uncemented tapered hip stems: a prospective 84-month follow-up study and a median 156-month cross-sectional study with DXA. Calcified Tissue International.

[3] Roth A (2005). [Periprosthetic bone loss after total hip endoprosthesis. Dependence on the type of prosthesis and preoperative bone configuration]. Orthopade.

[4] Bodén HSG, Sköldenberg OG, Salemyr MOF, Lundberg H-J, Adolphson PY (2006). Continuous bone loss around a tapered uncemented femoral stem: a long-term evaluation with DEXA. Acta Orthop.

[5] Stukenborg-Colsman CM (2012). Bone remodelling around a cementless straight THA stem: a prospective dual-energy X-ray absorptiometry study. Hip Int.

[6] Kröger H (1997). Periprosthetic bone loss and regional bone turnover in uncemented total hip arthroplasty: a prospective study using high resolution single photon emission tomography and dual-energy X-ray absorptiometry. J. Bone Miner. Res..

[7] Eckardt A, Karbowski A, Schwitalle M, Herbsthofer B, Kreitner KF (1997). [Radiological changes after implantation of 2 different cementless hip prostheses]. Rofo.

[8] Merle C (2011). Bone remodeling around stable uncemented titanium stems during the second decade after total hip arthroplasty: a DXA study at 12 and 17 years. Osteoporos Int.

[9] Alm JJ (2009). Female patients with low systemic BMD are prone to bone loss in Gruen zone 7 after cementless total hip arthroplasty. Acta Orthop.

[10] Venesmaa P, Vanninen E, Miettinen H, Kröger H (2012). Periprosthetic bone turnover after primary total hip arthroplasty measured by single-photon emission computed tomography. Scand J Surg.

[11] Karachalios T (2004). The long-term clinical relevance of calcar atrophy caused by stress shielding in total hip arthroplasty: a 10-year, prospective, randomized study. J Arthroplasty.

[12] Spotorno L (1993). The CLS system. Theoretical concept and results. Acta Orthop Belg.

[13] Khanuja HS, Vakil JJ, Goddard MS, Mont MA (2011). Cementless femoral fixation in total hip arthroplasty. J Bone Joint Surg Am.

[14] Stihsen C, Radl R, Keshmiri A, Rehak P, Windhager R (2012). Subsidence of a cementless femoral component influenced by body weight and body mass index. Int Orthop.

[15] Hagel A, Hein W, Wohlrab D (2008). Experience with the Mayo conservative hip system. Acta Chir Orthop Traumatol Cech.

[16] Gruen, T. A., McNeice, G. M. & Amstutz, H. C. ‘Modes of failure’ of cemented stem-type femoral components: a radiographic analysis of loosening. *Clin. Orthop. Relat. Res*. 17–27 (1979). 477100

[17] Ruff CB, Hayes WC (1988). Sex differences in age-related remodeling of the femur and tibia. J. Orthop. Res..

[18] Reiter A (1997). [Periprosthetic mineral density in cement-free hip replacement arthroplasty]. Z Orthop Ihre Grenzgeb.

[19] Sessa G (2019). Bone mineral density as a marker of hip implant longevity: a prospective assessment of a cementless stem with dual-energy X-ray absorptiometry at twenty years. Int Orthop.

[20] Yukizawa Y (2017). Efficacy of Alendronate for the Prevention of Bone Loss in Calcar Region Following Total Hip Arthroplasty. J Arthroplasty.

[21] Zerahn B, Borgwardt L, Ribel-Madsen S, Borgwardt A (2011). A prospective randomised study of periprosthetic femoral bone remodeling using four different bearings in hybrid total hip arthroplasty. Hip Int.

